# Multimodal phenotyping of foveal hypoplasia in albinism and albino-like conditions: a pediatric case series with adaptive optics insights

**DOI:** 10.1038/s41598-024-66326-0

**Published:** 2024-07-04

**Authors:** Giacomo M. Bacci, Elisa Marziali, Sara Bargiacchi, Michel Paques, Gianni Virgili, Pina Fortunato, Marine Durand, Camilla Rocca, Angelica Pagliazzi, Viviana Palazzo, Lucia Tiberi, Debora Vergani, Samuela Landini, Angela Peron, Rosangela Artuso, Bianca Pacini, Monica Stabile, Andrea Sodi, Roberto Caputo

**Affiliations:** 1 Pediatric Ophthalmology Unit, Meyer Children’s Hospital IRCCS, Viale Pieraccini 24, 50139 Firenze, Italy; 2 Medical Genetics Unit, Meyer Children’s Hospital IRCCS, Florence, Italy; 3 Clinical Investigation Center Vision 1423, INSERM-DGOS, Sorbonne Université, Quinze-Vingts Hospital, Paris, France; 4 Institut de la Vision, Paris, France; 5 Department of Neuroscience, Psychology, Drug Research and Child Health, University of Florence, Florence, Italy; 6 IRCCS – Fondazione Bietti, Rome, Italy; 7 Imagine Eyes, Orsay, France; 8 Department of Biomedical Experimental and Clinical Sciences “Mario Serio”, University of Florence, Florence, Italy; 9 Nephrology and Renal Transplantation Research Group, KU Leuven, Leuven, Belgium; 10 Department of Clinical and Experimental Medicine, Section of Pediatrics, University of Pisa, Pisa, Italy

**Keywords:** Foveal hypoplasia, Adaptive optics, Cone mosaic, OCT, OCT-A, *TYR*, OCA, Paediatric research, Medical genetics, Disease genetics

## Abstract

Aim of the present study is to evaluate the relationship between genetic and phenotypic data in a series of patients affected by grade I and II of foveal hypoplasia with stable fixation and good visual acuity using multimodal imaging techniques. All patients underwent complete clinical and instrumental assessment including structural Optical Coherence Tomography (OCT), OCT Angiography and Adaptive Optics (AO) imaging. Central macular thickness (CMT), inner nuclear layer (INL), vessel density in superficial capillary plexus were the main variables evaluated with OCT technology. Cone density, cone spacing, cone regularity, cone dispersion and angular density were the parameters evaluated with AO. Genetic evaluation and trio exome sequencing were performed in all affected individuals. Eight patients (3 males and 5 females) with a mean age of 12.62 years (range 8–18) were enrolled. The mean best corrected visual acuity (BCVA) was 0.18 ± 0.13 logMAR, mean CMT was 291.9 ± 16.6 µm and INL was 26.2 ± 4.6 µm. The absence of a foveal avascular zone (FAZ) was documented by examination of OCT-A in seven patients in the superficial capillary plexus. However, there was a partial FAZ in the deep plexus in patients P5 and P8. Of note, all the patients presented with major retinal vessels clearly crossing the foveal center. All individuals exhibited a grade I or II of foveal hypoplasia. In 5 patients molecular analyses showed an extremely mild form of albinism caused by compound heterozygosity of a *TYR* pathogenic variant and the hypomorphic p.[Ser192Tyr;Arg402Gln] haplotype. One patient had Waardenburg syndrome type 2A caused by a de novo variant in *MITF.* Two patients had inconclusive molecular analyses. All the patients displayed abnormalities on OCT-A. Photoreceptor count did not differ from normal subjects according to the current literature, but qualitative analysis of AO imaging showed distinctive features likely related to an abnormal pigment distribution in this subset of individuals. In patients with foveal hypoplasia, genetic and multimodal imaging data, including AO findings, can help understand the physiopathology of the foveal hypoplasia phenotype. This study confirms that cone density and visual function can both be preserved despite the absence of a pit.

## Introduction

Foveal hypoplasia^1^ is a condition characterized by absence of the foveal pit, reduced macular pigment and absence of the foveal avascular zone (FAZ)^2,3^. Traditionally, the absence of the foveal pit has been considered the main reason of poor visual acuity in patients affected by FH, even though the role of the foveal pit on visual function has been questioned^4^. The primary role of genetic and non-genetic factors in the development of FH has already been described^5,6^. Although recent genetic findings provide new insight on this condition, FH has also been described to possibly occur as an isolated form (isolated foveal hypoplasia) or associated with inherited diseases such as albinism, aniridia, achromatopsia, microphthalmos, incontinentia pigmenti, optic nerve hypoplasia, choroideremia, familial exudative vitreoretinopathy, and Stickler syndrome^7^. The association between FH and non-genetic conditions such as retinopathy of prematurity and high myopia has also been reported^8–10^: a single case describing foveal hypoplasia associated to congenital rubella pose the question that other causes but genetics can perturb foveal development.

The clinical identification of FH can be challenging, especially in young children, because retinal findings that are clearly detectable on simple fundus examination are often missing. Moreover FH can be completely asymptomatic or mildly symptomatic in children^11^. In recent years, FH has been studied using different retinal imaging modalities such as fluorescein angiography^4^ (FA), optical coherence tomography (OCT), and optical coherence tomography angiography (OCT-A)^12^. Given the accuracy and definition of OCT images, a simplified grading system based on tomographic findings has been developed^13,14^ and is routinely used in clinical practice. This grading system is based on different stages of halted development of the fovea, in particular the presence or absence of the foveal pit, and widening of the outer nuclear layer^15^ and outer segment (OS)^15^. Several OCT-A-based studies have shown absence of the FAZ in the superficial and deep capillary plexus (SCP and DCP) in patients with FH^16,17^. In the last decade, adaptive optics (AO) imaging has emerged as a revolutionary imaging technique, allowing imaging of the living retina at a cellular resolution, and has been rapidly changing our understanding of retinal diseases. However, a limited number of studies have applied AO—together with genotypic information—to investigate the characteristics of cone layout and cone distribution in pediatric patients affected by FH^4^.

The aim of this study was to characterize a series of children with clinically and genetically determined FH using a multimodal approach that includes AO imaging, Swept-source-OCT (SS-OCT) and OCT-A. The main advantage of these imaging technologies is their complementarity to study structural retina, angiographic and photoreceptors features. In particular, AO focuses mainly on the outer retina and OCT-A mainly on the inner retina. Here we demonstrate that specific characteristics on AO and OCT-A in paediatric patients may yield new insight into the physiopathology of FH and its related genotype. 

## Methods

This is a prospective monocentric study carried out from August 2019 to March 2020 at the Paediatric Ophthalmology Department of Meyer Children’s Hospital IRCCS in Florence, Italy. We included patients with a clinical diagnosis of FH to explore and describe their phenotypic and genotypic characteristics.

Inclusion criteria were: age at referral between 6 and 18 years; FH diagnosed by fundus examination (absence of foveal reflex) and confirmed through SS-OCT and OCT-A (partial loss of the foveal pit on structural OCT and abnormalities of the FAZ on OCT-A); best corrected visual acuity (BCVA) better than 0.5 logMAR in both eyes; and the ability to maintain moderately steady fixation with both eyes. The main exclusion criteria were: presence of nystagmus precluding proper instrumental analysis and any other ophthalmologic or systemic disease that could affect reliability of the measurements. Spherical refraction of more than ± 6 diopters (D) or cylinder refraction of ± 2 D and any central media opacity sufficient to hinder SS-OCT, OCT-A and AO examinations were also considered exclusion criteria due to the poor quality of in-depth examination.

All patients underwent genetic evaluation by a clinical geneticist trained in ocular genetics and complete pediatric ophthalmological assessment including cycloplegic refraction, BCVA obtained in logMAR Unit using age-appropriate ETDRS charts, saturated Panel D-15 color vision test (The Good-Lite Company, Elgin, IL), anterior segment biomicroscopy and dilated indirect fundus examination. Retinal imaging procedures were performed after pupil dilation and cycloplegic refraction. In all affected individuals, axial length was measured using an interferometer (IOL Master; Carl Zeiss Meditec, Dublin, CA).

### Molecular analysis

Genomic DNA of all the patients enrolled at the Division of Medical Genetics of Meyer Children’s Hospital IRCCS, Florence (Italy) was extracted from peripheral blood using the QIAamp Mini Kit (QIAGEN®, Hilden, Germany) and was used to build libraries (Kapa Biosystems, Wilmington, MA) for trio exome sequencing (ES). Captured libraries obtained using the protocol SeqCap EZ Exome v3, (Nimblegen, Roche, Basel, Switzerland) were sequenced with NextSeq500/550 (Illumina Inc., San Diego, CA, USA).

The FASTQ files produced by the sequencer were elaborated using a bioinformatic automated in-house pipeline, enabling reads alignment to the reference hg19 genome (Burrows–Wheeler Aligner, BWA), mapping and analysis with the Integrative Genome Viewer (IGV) software^18^, variant calling (Genome Analysis ToolKit Unified Genotyper Module, GATK)^19^ and variant annotation (Annovar)^20^ as previously described^21,22^.

The variant prioritization strategy followed these filtering criteria:

- mode of inheritance (*de-novo*, autosomal dominant, autosomal recessive and X-linked);
- variants with no frequency data or with a minor allele frequency (MAF) ≤ 0.01 for genes with autosomal recessive (AR) inheritance and with a MAF ≤ 0.001 for genes with autosomal dominant (AD) inheritance, according to gnomAD https://gnomad.broadinstitute.org) and the laboratory in-house exome control cohort (3000 exomes) of unrelated individuals analyzed for non-ocular diseases;
- non-synonymous, short insertion/deletion, synonymous or splice-site variants (20 bp splice acceptor, 20 bp splice donor sites);
- variants in genes associated with FH reported in Online Mendelian Inheritance in Man (OMIM), Human Gene Mutation Database (HGMD), Human Phenotype Ontology (HPO) or in the scientific literature revised on December 22, 2023; (Supp. Table S1)
- manual inspection for the p.(Arg402Gln) and p.(Ser192Tyr) variants in *TYR*^23^.

The filtered variants were evaluated considering disease-causing mutation database (ClinVar, HGMD) and in silico preditiction tools (MetaRNN, CADD, Polyphen-2, SIFT, Mutation Taster, MutationAssessor, FATHMM, FATHMM MKL, SpliceAI, ADA score). The prioritised variants were classified according to the American College of Medical Genetics (ACMG) guidelines^24^.

Moreover, we implemented our in-house pipeline with a normalized read count approach to determine the presence of copy number variations (CNVs) considering only selected genes as previously described^21^. All the identified variants were confirmed by Sanger sequencing.

### OCT procedure

Images of the macula were obtained using 6 × 6 mm scans (3DMacula H) with SS-OCT (Topcon 3D, DRI OCT Triton, Topcon Corporation, Tokyo, Japan). The internal fixation target of the system was used. Focus of the fundus image was optimized using built-in focus correction and the polarization setting was optimized using the built-in function. Retinal thickness data from the macular volume scans and the location of the foveal pit’s center were exported. At least three replicate scans were acquired to ensure repeatability of fixation. On OCT images inner limiting membrane (ILM), external limiting membrane (ELM), inner segment-outer segment junction (IS/OS), and the retinal pigment epithelium (RPE) were manually segmented. The distance between the ILM and the RPE provided the central macular thickness (CMT), which was manually determined. The thickness of the central inner nuclear layer (INL) was manually measured. To detect the actual center of the fovea we used a co-localization procedure thanks to multimodal imaging device: combining and overlapping, with the built-in software of SS-OCT, structural images with fundus photography and OCT-A we were able to manually detect the central macular area and to proceed measuring the CMT; additionally, we overlapped the AO montage to detect the central macular area also in AO images (Fig. 3). The different degrees of FH were classified according to the Leicester classification system proposed by Thomas et al.^13^

OCT-A images were obtained with Swept Source OCT-A (Topcon 3D, DRI OCT Triton, Topcon Corporation, Tokyo, Japan). The standard images obtained included 3 × 3 mm macular scans. Automated instrument segmentation parameters were used to define the superficial and the deep capillary plexus, specifically from 3 µm below the internal limiting membrane to 15 µm below the inner plexiform layer^25^ (superficial capillary plexus), and between 15 and 70 µm below the IPL (deep capillary plexus), respectively. Further manual segmentation was used to confirm that the presence or absence of the FAZ was not due to automated segmentation errors. Vessel density (VD) of the superficial capillary plexus (SCP) was obtained automatically using the DRI-OCT Triton software.

### Adaptive optics procedure

A series of images of the retina were acquired on each eye using a commercially available flood-illuminated AO retinal camera (rtx1™, Imagine Eyes, Orsay, France). AO imaging sessions were conducted using cycloplegic eye drops. The spherical ametropia was adjusted after entering the patient’s spheric equivalent refractive error. The patient was asked to stare at a yellow cross, controlled by the operator. Images of the macula were acquired, with the fixation target first at the center, then moved to 2° of eccentricity along the four meridians (nasal, temporal, superior and inferior). Each AO image covered a 4° × 4° field-of-view.

Cone mosaic metrics (local density, spacing, regularity and dispersion) were analyzed at 2° of eccentricity temporally, superiorly, nasally and inferiorly. To locate the foveal center, the overlapping AO images were registered by an automated mosaicking software (i2k Retina AO, Dual Align, USA) to obtain a montage, which was manually colocalized on OCT-A and OCT images of the macula. The eccentricities for analysis were then determined by measurements on the AO montage. Cones were automatically detected by the segmentation software provided by the manufacturer (AOdetect Mosaic V.3.0, Imagine Eyes, France) in Regions Of Interest (ROI) of 80 × 80 pixels, located at the previously defined eccentricities. The ROI size corresponds to 62 × 62 μm on the retina for an eye of 24 mm axial length. When the ROI fell on a vessel shadow, it was slightly shifted towards a measurable area according to best practice^26^. A manual correction of the automatic cone detection was performed by an experienced investigator at each retinal eccentricity. Cone density, cone spacing, regularity, dispersion and cone arrangement were computed by AO detect, considering the axial length of the eye. The angular density was measured as well.

This study adhered to the principles of the Declaration of Helsinki and was approved by the Pediatric Ethics Committee of Tuscany n° 135/2019. The patients’ legal guardians signed written informed consent.

## Results

A total of 8 patients met the inclusion criteria. Eight patients (16 eyes) with FH (from 7 unrelated families) were included in the study: 3 males and 5 females. Mean (± SD) age of the patients at referral was 12.62 (± 3.62 SD) years, ranging 8–18 years. All the patients were of Caucasian ethnicity and in none of them was a history of prematurity reported.

Family history was unremarkable in all the patients. Clinical evaluation showed isolated ophthalmological problems in all affected individuals, except for patient P5, who also exhibited bilateral sensorineural hearing loss.

All the patients showed the same degree of FH in both eyes (grade 1 or grade 2 according to Leicester classification^13,27^). Phenotypic data are summarized in Table 1. Patients P2, P6 and P7 have been previously reported in Rocca et al., 2022 and their phenotype is updated here.

**Table 1:** Phenotypic data and Genetic findings: Reported patients with variants in *TYR*(NM_000372.5), *MITF*(NM_000248.4) and *OCA2*(NM_000275.3).

Proband (sex)	Age (yrs)	FH Grading	BCVA RE	BCVA LE	Allele 1	GnomAD v2.1.1 (MAF General Population)	ACMG Classification	Allele 2	Reference
P1 (F)	14	1	0.22	0.3	*TYR*: c.1037-7 T > A;p.?	0.0008614	Pathogenic (PS3,PP3,PP5, PM2)	TYR: c.1205G > A;p.R402Q TYR: c.575C > A;p.S192Y	(Spritz et al.,1993)
P2 (M)	8	2	0	0	TYR: c.1205G > A;p.R402Q *TYR*: c.455C > G;p.P152R	n.a	Likely Pathogenic (PM1,PM2,PM5)	TYR: c.1205G > A;p.R402Q TYR: c.575C > A;p.S192Y	(Rocca et al., 2022)
P3 (M)	11	1	0.22	0.1	*OCA2*: c.2245-6C > A;p.?	0.0001018	VUS (PM2,PP5,BP4)	OCA2:c.1256G > A;p.R419Q MC1R: c.451C > T;p.R151C	(Mauri et al., 2017)
P4 (M)	11	2	0.3	0.22	TYR: c.1205G > A;p.R402Q *TYR*: c.739 T > C;p.C247R	n.a	Pathogenic (PM1,PM2,PP3, PP5)	TYR: c.1205G > A;p.R402Q TYR: c.575C > A;p.S192Y	(Urtatiz, Sanabria, and Lattig, 2014)
P5 (F)	9	1	0	0	*MITF*: c.808C > T;p.R270*	n.a	Pathogenic (PVS1,PP5,PM2)	Wild type	(Sun et al., 2016)
P6 (F) P7 (F)	18 16	2 2	0.3 0.22	0.22 0.22	*TYR*: c.140G > A;p.G47D	0.0001556	Pathogenic (PM1,PM2, PM5,PP5)	TYR: c.1205G > A;p.R402Q TYR: c.575C > A;p.S192Y	(Marti et al.,2018;Oetting et al., 1993)
P8 (F)	14	1	0.4	0.1	TYR: c.575C > A;p.S192Y *OCA2*:c.1076G > A;p.G359D	0.000007964	Pathogenic (PM1,PM2, PM5,PP3,PP5)	TYR: c.575C > A;p.S192Y OCA2:c.1065G > A;p.A355 =	(Mauri et al., 2017)

Frequency data refer to the GnomAD v2.1.1 General Population. Polymorphisms are shown in bold: *TYR*: c.1205G > A (MAF: 0.1765), *TYR*: c.575C > A (MAF: 0.2502), *OCA2*:c.1256G > A (MAF: 0.04658), *MC1R*: c.451C > T (MAF: 0.04481), *OCA2*:c.1065G > A (MAF: 0.6353). The table shows frequency data and the ACMG classification for the rare variants. The results of segregation are discussed in the text. In P3 and P8 molecular results were inconclusive.

Overall mean BCVA was 0.18 ± 0.13 logMAR. The overall mean CMT was 291.9 ± 16.6 µm and the overall mean INL was 26.2 ± 4.6 µm.

The absence of a FAZ was documented by examination of OCT-A in seven patients. We excluded one patient from OCT-A analysis due to the poor quality of the OCT-A image. On OCT-A, no other specific abnormalities were noticed in the outer retina or choriocapillaris. There was no evidence of a FAZ in the superficial capillary plexus. However, there was a partial FAZ in the deep plexus in patients P5 and P8. Of note, all the patients presented with major retinal vessels clearly crossing the foveal center. The vessel density in the superficial plexus obtained by OCT-A was 40.8% ± 5.28%.

AO imaging allowed for quantitative and qualitative data analysis. The quantitative results are displayed in Table 2. In all of our patients a qualitative evaluation of AO imaging showed the presence of diffuse “pigmentary abnormalities” in the macular area and the same distribution of photoreceptor in all the macular areas without the characteristic blurred area that is present in healthy subject.

**Table 2:** BCVA (Best Correct Visual Acuity), CMT (Central Macular Thickness), INL (Inner Nuclear Layer), cone density, cone spacing, cone regularity, cone dispersion, angular density (measured at 2° eccentricity from the fovea in the 4 quadrants) (reference for normative values (Sharma, 2023 #2851) and vessel density (measured on OCTA) in the study group.

Parameter		Total
BCVA		0.18 ± 0.13
CMT		291.9 ± 16.6
INL		26.2 ± 4.6
VD SCP		40.8 ± 5.28
Density 2° (metric-mm^2^)	Nasal	28,425 ± 4619
	Superior	24,963 ± 3687
	Inferior	25,682 ± 3139
	Temporal	27,262 ± 5905
Spacing 2°(metric-micron)	Nasal	6.62 ± 0.56
	Superior	7.02 ± 0.53
	Inferior	6.88 ± 0.51
	Temporal	6.80 ± 0.83
Regularity 2° (%)	Nasal	94.70 ± 2.91
	Superior	94.13 ± 3.85
	Inferior	95.75 ± 4.13
	Temporal	96.77 ± 2.80
Dispersion 2° (%)	Nasal	10.64 ± 2.62
	Superior	12.04 ± 4.28
	Inferior	10.2 ± 2.55
	Temporal	9.73 ± 2.27
Angular density 2°	Nasal	1953 ± 305
	Superior	1764 ± 134
	Inferior	1838 ± 158
	Temporal	1902 ± 306

The results of the analyses are expressed as mean ± SD for quantitative variables.

**Figure 1:**
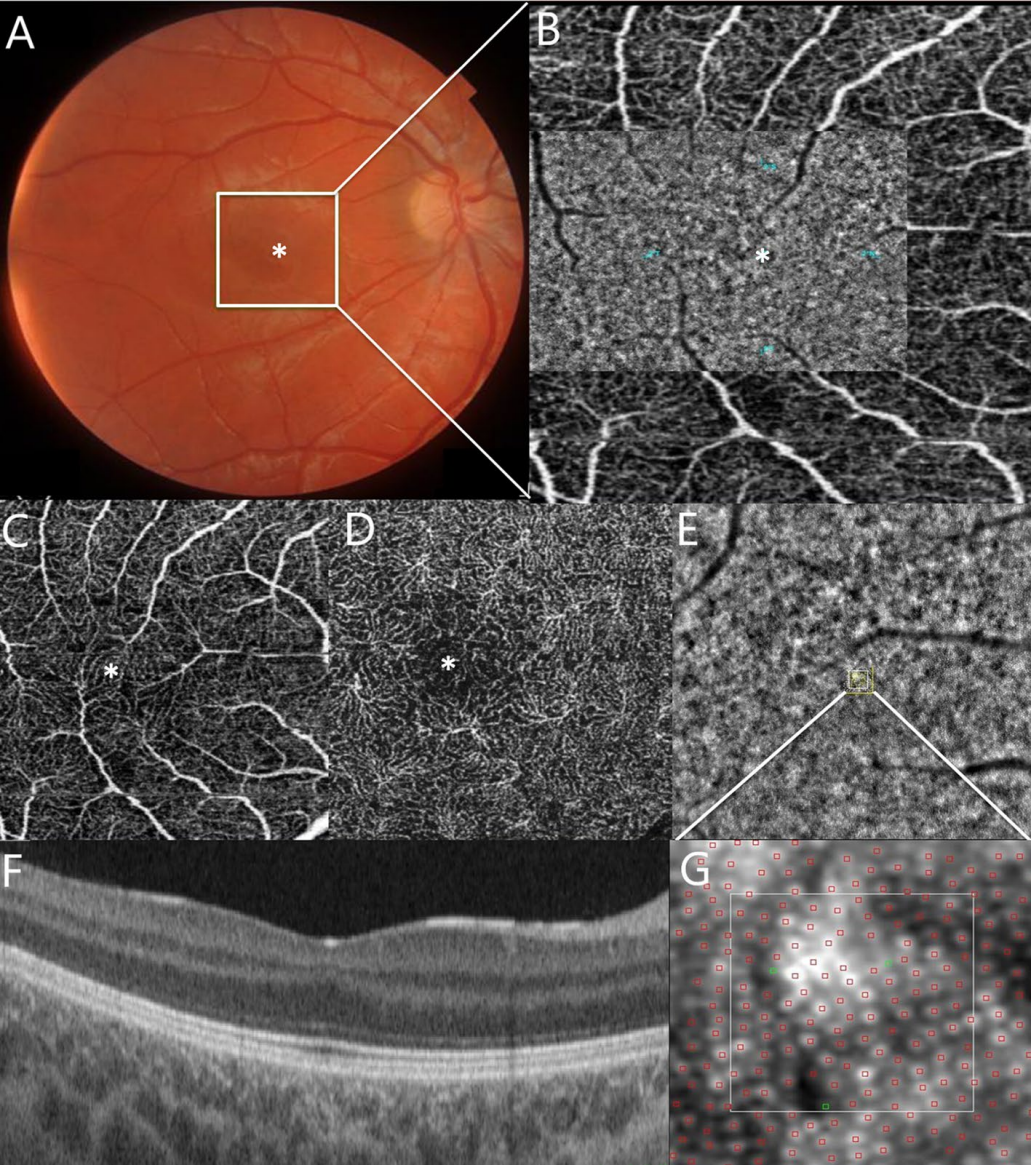
Composite imaging showing a sample of acquisition and interpretation of data. (**A**) Color fundus photography of the RE of a 15-year-old female affected by grade 1 foveal hypoplasia (P8F). The image in the white square is magnified in B. (B) 3 × 3 mm OCTA of the SCP of the patient’s RE. (**B**) Flood-illuminated Adaptive Optics (FIAO) image montage overlaid on the OCTA image of the SCP demonstrates the appearance of the cone mosaic. The cone counting was measured at 2° eccentricity from the fovea in four meridians: nasal, temporal, superior and inferior (blue dots). (**C**) SCP and (**D**) DCP showing the lack of the fovea avascular zone (FAZ) on OCT angiography. (**E**) FIAO imaging of the cone mosaic nasally to the fovea showing the appearance of the cone mosaic. The yellow square is magnified in G and represents the Region Of Interest (ROI) where the cone mosaic was counted. This area is located at 2° eccentricity from the fovea in the nasal area. **(F)** Structural OCT showing persistence of the inner retinal layer and the presence of a widening of the outer nuclear layer^15^. (**G**) Magnification image of the yellow square in picture E shows the cone counting analyzed using the software package AO Detect Mosaic V.3.0. The manufacturer’s software automatically detects the cone mosaic and the position of the photoreceptors; it also enables manual correction.

**Figure 2:**
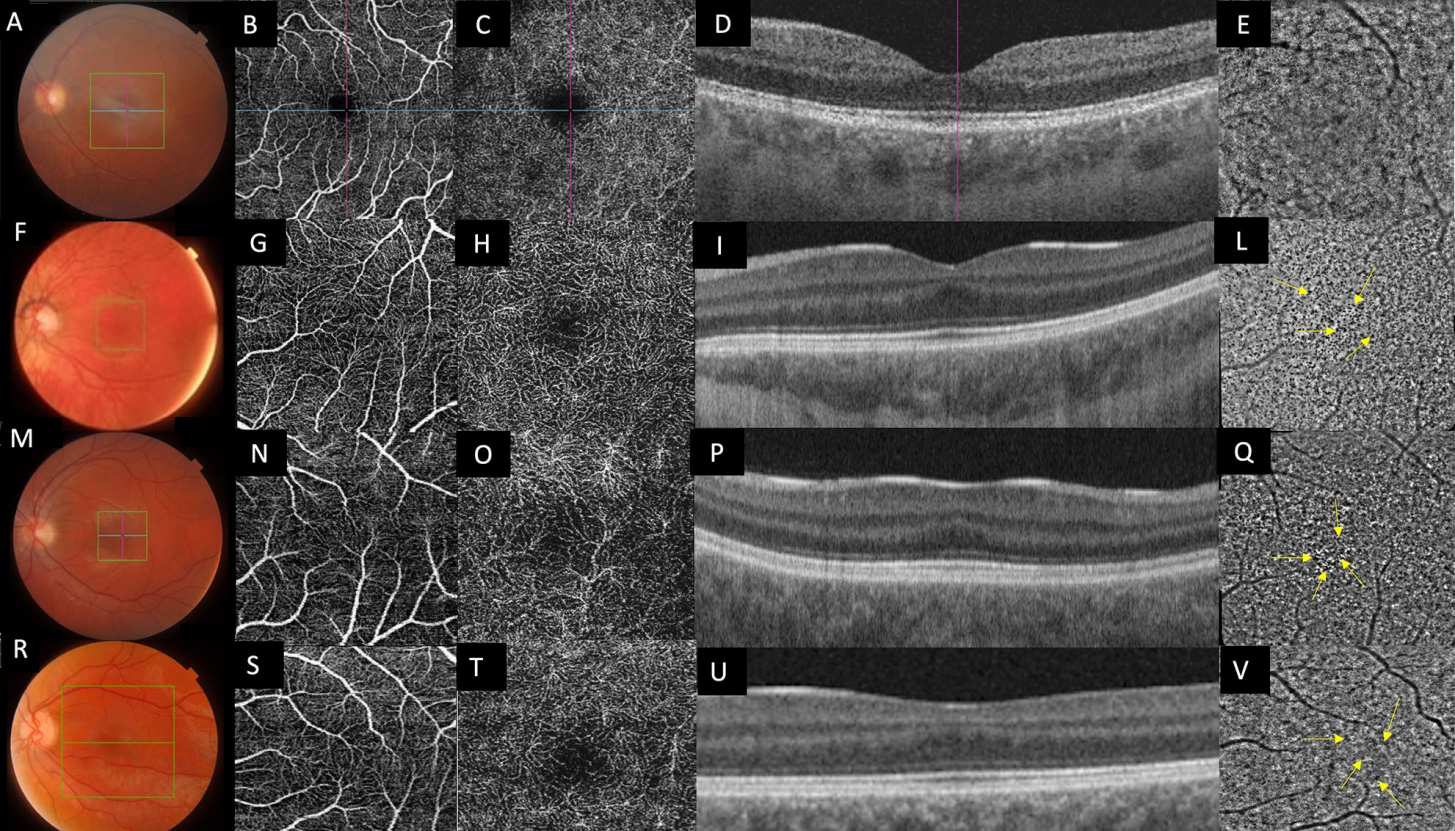
Composite imaging of multimodal imaging of FH: (**A**–**E**): normal control**.** (**F**, **M**, **R**) Color fundus photography of the LE of patients affected by foveal hypoplasia**:** corresponding OCT-A examinations of the SCP (**G**, **N**, **S**) and DCP (**H**, **O**, **T**). Cross sectional SS-OCT passing through the fovea (**I**, **P**, **U).** (**L**, **Q**, **V**) AO Imaging showing the appearance of the cone mosaic at the macula. The cone photoreceptors appear as ‘bright’ hyper-reflective dots over either a hyporeflective or isoreflective background: to ease the view, the image of the little red square was zoomed in the big red square on the bottom right corner of each AO report. Yellow arrows indicate the “pigmentary abnormalities” (see text).

To qualitatively compare the multimodal examinations of different degrees of FH, although related to different mutations, we present examples of the various phenotypes from the mildest to the most severe degree of FH based on the structural features investigated with SS-OCT and OCT-A. This approach permitted also an evaluation of AO characteristics for comparison with a healthy subject (Figs. 1 and 2).

The results of molecular analyses are summarized in Table 1. Five patients were diagnosed with an extremely mild form of albinism caused by compound heterozygosity of a *TYR* pathogenic variant and the hypomorphic p.[Ser192Tyr;Arg402Gln] haplotype^22,28,29^. One patient had Waardenburg syndrome type 2A, caused by a de novo variant in *MITF.* Two individuals had inconclusive results.

We did not find any significant differences in AO images among patients affected by FH with different genetic diagnoses (Fig. 2).

## Discussion

Our patients were initially evaluated for a moderate reduction of BCVA attributed only to a possible isolated FH, and their actual phenotype was reinterpreted correctly only after molecular testing identified a mild form of albinism in five patients and a syndromic form in another one. Only two affected individuals had inconclusive molecular result, but the presence of a pathogenic variant in known albinism genes or other conditions with retinal findings consistent with FH not detectable by exome sequencing cannot be ruled out. For instance, these patients could have a deep-intronic *OCA2* variant *in trans*, which cannot be detected by exome sequencing, or their phenotype could result from a complex haplotype, as for the *TYR* gene, or from digenic inheritance.

This study, although with a small number of patients, confirms that variants in *TYR* represent the most common causal association for FH, not only when FH is part of albinism but also when FH is clinically isolated^22,27^.

Foveal hypoplasia, and its relationship with albinism, is a condition of great interest with many aspects that have yet to be elucidated since the precise mechanisms causing the lack of development of the foveal pit has not been fully understood yet. However, the recent literature is starting to shed light on the clinical significance of FH and its role in understanding retinal development and plasticity^5,27,30^. The FAZ morphology is thought to be one of the main diagnostic clues in understanding the development of the foveal pit and the final foveal structure^31^. A distinct FAZ has been reported as early as gestational age 25 weeks^32^. While in the past it was thought that the FAZ was vascularized at some point, more convincing evidence suggests that it is always avascular during development^32,33^. When it fails to form, the foveal pit also fails to form. Moreover, recent evidence indicates that the identification of a FAZ could help distinguish between FH related to a disrupted development (i.e. oculocutaneous albinism), where the FAZ is absent, and FH related to retinal dystrophy (i.e. achromatopsia), where it is usually preserved^27,34^. In addition, a larger FAZ seems to be associated with a larger foveal pit^21,35^.

In recent years, several studies performed a thorough examination of normal foveal development including extrusion of the plexiform layers, outer segment lengthening and outer nuclear layer widening^5,36^. It is now well established that foveal hypoplasia may be present in individuals with a spectrum of visual acuities ranging from good to poor^37–39^. Nevertheless, how the morphological variability associated with foveal hypoplasia relates to visual prognosis remains unclear, as well as which specific features at the fovea may be more important in determining the visual performance. Several years ago, the term “fovea plana” was used to describe patients affected by FH. Indeed, the term FH may imply a retinal dysfunction, but this is not always the case^3^. Nevertheless, even “fovea plana” is not a good term to describe this clinical feature, since there is rarely a complete flat fovea (absence of foveal pit), and therefore a definition based on the size of the foveal avascular zone may be preferable: it could be named "small FAZ" eyes, but we recognize that such definition would require a consensus proposal and studies on hundreds of patients unlike the present study.

Some Authors suggested that the degree of foveal hypoplasia could be a predictor of the best corrected visual acuity^13^. Marmor et al.^4^ reported that in an adult population with good visual acuity and an absent foveal pit, the foveal cone specialization can be preserved both anatomically and functionally: the authors described subjects with cones of normal diameter in the central macula on adaptive optics imaging. Moreover, Noval et al.^11^ reported that the incidence of FH detected by OCT in a cohort of healthy children is 1.7–3%: although interesting, in their series the authors reported only grade 1 or 2 foveal hypoplasia. Rufai et al*.*^40^ tried to correlate the macular morphology as a predictor of visual function, but they evaluated only patients with albinism, infantile idiopathic nystagmus and achromatopsia, while Harvey et al*.*^39^ showed a weak but significant correlation between macular thickness and visual acuity. In contrast, Holmstrom et al*.*^41^ did not find any significant correlation between the central macular thickness and visual acuity.

**Figure 3:**
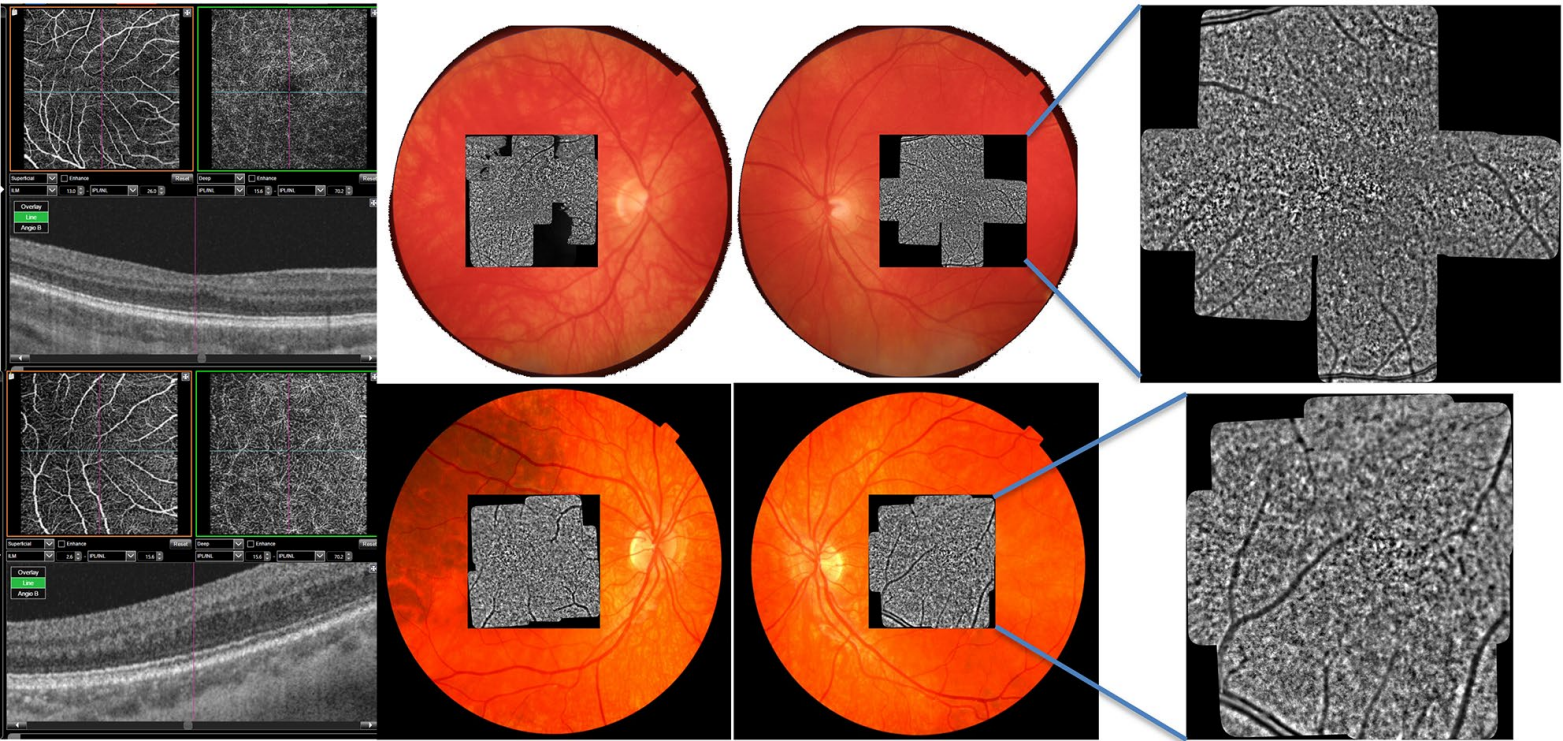
Composite of OCT-A, fundus photography and AO imaging of patients P1 and P2. 1A: note absence of the FAZ in OCT-A and a grade 1 of FH. 1B: Fundus photography with overlapping AO (see text). The multi modal imaging, overlapping AO cone mosaic to fundus photograph and OCT and OCT-A imaging, allowed us to detect the actual central foveal area also in AO.

From a morphological point of view, a qualitative evaluation of Adaptive Optics imaging evidenced the presence of diffuse “pigmentary abnormalities” in the macular area in all patients, whose origin is unknown (Fig. 2). This finding is detectable in all patients regardless of different genotypes and it could represent an interesting finding, if compared to healthy subjects, possibly related to the identification of retinal melanosomes. The melanosome is a lysosome related organelle, where melanin is produced and stored, representing the intracellular site of pigmentation in a melanocyte or RPE cell. Since it is well known that in syndromic and non-syndromic forms of albinism the retinal pigmentation pathway is altered^42^, one may argue that adaptive optics imaging could have the potential to investigate the abnormal distribution of retinal pigmentation allowing new insight into pathogenic mechanisms of disrupted embryogenesis in albinos and albino-like phenotypes. This observation has limitation since melanin is also present in the choroid and the RPE. Moreover, the strong background illumination alters the usual landmarks for interpretation, raising the issue of interpretating AO data, which remains perfectible. Another interesting morphological finding clearly detectable through AO in our series, if compared with healthy subject, is the absence of a central blurring in the foveal zone (Fig. 2). In fact, photoreceptors are packed in the normal fovea due to their centripetal migration during retina development^5^: this characteristic hinders proper visualization of the photoreceptors in the foveal area with flood-illuminated adaptive optics (Fig. 3). In patients with foveal hypoplasia also the photoreceptors in the foveal area could be correctly identified suggesting a lower than normal packing^4^. The cone packing at the fovea was reduced in our patients: it is usually impossible to measure cone number in the fovea because of massive cone packing in healthy subjects, but in our patients we were able to count the cones, thus determining that they are less packed. Values of foveal cone density from rtx1 images on the normal fovea (regardless patients’ age) cannot be found in other publications since it cannot be measured. However, normal cone density values measured with rtx1, at 2° on four quadrants in *young* subjects have been recently reported by Sharma et al.^43^.

Moreover, previous studies have qualitatively discussed an abnormally low cone density at the fovea based on the comparison between rtx1 images in patients affected by Stargardt disease vs. controls^44^, achromatopsia^45^, and Bestrophinopathy^46^.

It was not surprising that the majority of our patients showed foveal hypoplasia related to mild albinism, in line with recent literature that considers different forms of albinism (isolated and syndromic) as a spectrum, instead of different clinical entities^22,47^. To the best of our knowledge, this is one of the first studies that describe an in-depth characterization, including adaptive optics, of a distinctive pediatric cohort affected by some degree of FH and related genotypes. In our series, the degree of foveal hypoplasia did not show differences in patients with different visual acuities. Noteworthy, our AO data at 2° of eccentricity are almost overlapping with the results of Legras et al.^48^, who focused on a population of healthy adults. If this is true, one can speculate that different peculiarities from the distribution of photoreceptors or their own function, can influence visual acuity such as, for instance, RPE anatomy, IS/OS lengthening, or a distinctive inner retinal architecture that cannot be explored in the present study. Our results also support the findings of Noval et al*.*: since photoreceptor’s density and packing at 2° did not differ from healthy subjects in our series, it is possible that some individuals of the general population exhibit a low degree (1 or 2 according to the authors) of isolated foveal hypoplasia without a significant decrease in visual function.

Notwithstanding, our data must be interpreted with caution since our study has major limitations. Firstly, the limited sample size precluded a proper statistical analysis of the data. Also, all the patients were evaluated in a clinical setting and only FH of grade 1 and 2 were included in order to obtain the most reliable data: since usually the worst visual acuities are associated with more unstable fixation (i.e. congenital nystagmus) as grade 3 or 4^13^, we cannot exclude that peculiarities reside in these latter groups. In fact, we exclusively selected patients with stable fixation and without nystagmus to obtain qualitatively good images, which represents an important bias because the parameters in patients with severe foveal hypoplasia were not evaluated. Finally, our results are based only on structural imaging modalities, and we do not have a control group: we cannot exclude that functional modalities, such microperimetry or multifocal ERG, may be useful to explore the functional aspects of the visual cells more in detail and better explain cases with lower visual function despite similar cone density.

In conclusion, our study reinforces the potential of new imaging techniques—like adaptive optics—and of genomic technologies to improve understanding of apparently isolated foveal hypoplasia. Larger studies and deeper phenotyping are warranted to increase the knowledge and improve anatomic-functional relationships in such a distinctive phenotype.

### Supplementary Information

Supplementary Information.

## Data Availability

The datasets generated during and/or analysed during the current study is provided within the manuscript and in supplementary information file.
