# Supplementary material for: Multimodal phenotyping of foveal hypoplasia in albinism and albino-like conditions: a pediatric case series with adaptive optics insights
Source: Sci Rep. 2024 Jul 4;14:15454. doi: 10.1038/s41598-024-66326-0 (PMC11224352; doi:10.1038/s41598-024-66326-0)
Supplement: Supplementary file 1 — Supplementary Information. [file 41598_2024_66326_MOESM1_ESM.pdf]

Multimodal phenotyping of foveal hypoplasia in albinism and albino-like conditions: a pediatric case series with adaptive optics insights

Bacci Giacomo Maria[1*] MD PhD, Marziali Elisa[1*] MD, Bargiacchi Sara [2] MD, Paques Michel[3,4] MD PhD, Virgili Gianni[5,6] MD, Fortunato Pina[1] MD PhD, Durand Marine[7], Rocca Camilla[8] MD, Pagliazzi Angelica[9] MD, Palazzo Viviana[2], Tiberi Lucia [2], Vergani Debora [2], Landini Samuela [2], Peron Angela[2,8] MD PhD, Artuso Rosangela [2] PhD, Pacini Bianca[5] MD, Stabile Monica[10] MD, Sodi Andrea[5] MD, Caputo Roberto[1] MD

1: Pediatric Ophthalmology Unit A. Meyer Children's Hospital IRCCS, Florence, Italy
2: Medical Genetics Unit, Meyer University Hospital IRCCS, Florence, Italy
3: Clinical Investigation Center Vision 1423, INSERM-DGOS, Sorbonne Université, Quinze-Vingts Hospital, Paris, France.
4: Institut de la Vision, Paris, France.
5: Department of Neuroscience, Psychology, Drug Research and Child Health, University of Florence, Florence, Italy.
6: IRCCS – Fondazione Bietti, Rome, Italy.
7: Imagine Eyes, Orsay, France
8: Department of Biomedical Experimental and Clinical Sciences "Mario Serio", University of Florence, Florence, Italy.
9: Nephrology and Renal Transplantation Research Group, KU Leuven, Belgium
10: Department of Clinical and Experimental Medicine, Section of Pediatrics, University of Pisa, Pisa, Italy
*These authors equally contributed

Gene panel	
AHR	NM_001621.5
AP3B1	NM_003664.5
AP3D1	NM_001261826.3
BLM	NM_000057.4
BLOC1S3	NM_212550.5
BLOC1S5	NM_201280.3
BLOC1S6	NM_012388.4
CACNA1A	NM_001127221.2
CACNA1F	NM_005183.4
CASK	NM_003688.3
DTNBP1	NM_032122.5
EDN3	NM_207034.3
EDNRB	NM_000115.5
FRMD7	NM_194277.3
GNAI3	NM_006496.4
GPR143	NM_000273.3
HPS1	NM_000195.5
HPS3	NM_032383.5
HPS4	NM_022081.6
HPS5	NM_181507.2
HPS6	NM_024747.6
KITLG	NM_000899.5
KIT	NM_000222.3
LRMDA	NM_032024.5
LYST	NM_000081.4
MANBA	NM_005908.4
MITF	NM_000248.4
MLPH	NM_024101.7
MYO5A	NM_000259.3
OCA2	NM_000275.3
PAX3	NM_181457.4
PAX6	NM_000280.6
RAB27A	NM_004580.5
SACS	NM_014363.6
SETX	NM_015046.7
SLC24A5	NM_205850.3
SLC38A8	NM_001080442.3
SLC45A2	NM_016180.5
SNAI2	NM_003068.5
SOX10	NM_006941.4
TULP1	NM_003322.6
TYRP1	NM_000550.3
TYR	NM_000372.5

Supplementary Table S1. Panel of genes associated with foveal hypoplasia and analysed by Exome Sequencing
